# Supplementary material for: Agent-based model predicts that layered structure and 3D movement work synergistically to reduce bacterial load in 3D in vitro models of tuberculosis granuloma
Source: PLoS Comput Biol. 2024 Jul 12;20(7):e1012266. doi: 10.1371/journal.pcbi.1012266 (PMC11288457; doi:10.1371/journal.pcbi.1012266)
Supplement: S1 Table — (DOCX) [file pcbi.1012266.s001.docx]

**S1** **Table.** Parameters descriptions for the parameters that are varied during calibration.

| Parameter | Description |
| --- | --- |
| **Bacteria** |  |
| *mtbInternalDoublingTime* | The length of time in hours it takes for an intracellular bacterium to divide. |
| *mtbExternalDoublingTime* | The length of time in hours it takes an extracellular bacterium to divide. |
| **Macrophages** |  |
| *activatedMacrophageProportion* | The proportion of macrophages that begin the simulation activated. |
| *baseKillingProbability* | The probability a resting macrophage will kill an intracellular bacterium in a single tick. |
| *activeKillingProbability* | The probability an activated macrophage will kill an intracellular bacterium in a single tick. |
| *basePhagocytosisProbability* | The probability a resting macrophage will phagocytose a bacterium in a single tick given there is a bacteria to phagocytose in the Moore neighborhood of the macrophage. |
| *activePhagocytosisProbability* | The probability an activated macrophage will phagocytose a bacterium in a single tick given there is a bacteria to phagocytose in the Moore neighborhood of the macrophage. |
| *phagocytosisThreshold* | How many intracellular bacteria inside a macrophage before the macrophage can no longer phagocytose. |
| *cellularDysfunctionThreshold* | How many intracellular bacteria inside a macrophage before the macrophage has cellular dysfunction, meaning it can no longer kill bacteria. |
| *nfkbSpan* | Length of time in hours NF-κB will stay activated after a cell receives the activating signal. |
| *TNFthresholdForNFkBActivation* | Number of TNF-α molecules in the local grid cube required for macrophage NF-κB activation. |
| *bacThresholdForNFkBActivation* | Number of bacteria in the Moore neighborhood required for macrophage NF-κB activation. |
| *stat1Span* | Length of time in hours STAT1 will stay activated after a cell receives the activating signal. |
| *IFNthresholdForStat1Activation* | Number of IFN-γ molecules in the local grid cube required for macrophage STAT1 activation. |
| *ActivatedMacrophageTNFSecretion* | Molecules of TNF-α secreted from an activated macrophage in one second. |
| *InfectedMacrophageTNFSecretion* | Molecules of TNF-α secreted from an infected macrophage in one second. |
| *macrophagePopulation_MaxLifespan* | Number of days a resting macrophage will live *in vitro*. |
| *macrophagePopulation_MaxActivatedLifespan* | Number of days an activated macrophage will live *in vitro*. |
| *baseMovementProbabilityMacro* | The probability a resting macrophage will move to an adjacent grid cube in a single tick. |
| *activatedMovementProbabilityMacro* | The probability an activated macrophage will move to an adjacent grid cube in a single tick. |
| **CD4+ T cells** |  |
| *fractionCD4* | The proportion of CD3+ T cells that are CD4+ when T cells are added to the simulation. |
| *fractionTBSpecific* | The proportion of CD4+ T cells that are TB specific when T cells are added to the simulation. |
| *activatedTBSpecificCD4Fraction* | The proportion of TB specific CD4+ T cells that are activated when T cells are added to the simulation. |
| *CD4ActivationProbability* | The probability of CD4+ T cell activation per tick given a random neighboring macrophage has interacted with the bacteria. |
| *CD4DeactivationProbability* | The probability of a CD4+ T cell deactivating per tick. |
| *ActivatedCD4TNFSecretion* | Molecules of TNF-α secreted from an activated CD4+ T cell in one second. |
| *ActivatedCD4IFNSecretion* | Molecules of IFN-γ secreted from an activated CD4+ T cell in one second. |
| *cd4PopulationDoublingTime* | The length of time in hours it takes an activated CD4+ T cell to proliferate. |
| *maximumCD4Generations* | The maximum number of times a single CD4+ T cell can proliferate. |
| *cd4Population_MaxLifespan*  *cd8Population_MaxLifespan* | Number of days a resting CD4+ or CD8+ T cell will live *in vitro*. |
| *cd4Population_ActivatedLifespan*  *cd8Population_MaxActivatedLifespan* | Number of days an activated CD4+ or CD8+ T cell will live *in vitro*. |
| *baseMovementProbabilityCD4*  *baseMovementProbabilityCD8* | The probability a resting CD4+ or CD8+ T cell will move to an adjacent grid cube in a single tick. |
| *activatedMovementProbabilityCD4*  *activatedMovementProbabilityCD8* | The probability an activated CD4+ or CD8+ T cell will move to an adjacent grid cube in a single tick. |
| **CD8+ T cells** |  |
| *CD8Fraction* | The proportion of CD3+ T cells that are CD8+ when T cells are added to the simulation. |
| *tbSpecificCD8Fraction* | The proportion of CD8+ T cells that are TB specific when T cells are added to the simulation. |
| *activatedTBSpecificCD8Fraction* | The proportion of TB specific CD8+ T cells that are activated when T cells are added to the simulation. |
| *CD8ActivationProbability* | The probability of CD8+ T cell activation per tick given a random neighboring macrophage has interacted with the bacteria and is STAT1 activated. |
| *CD8DeactivationProbability* | The probability of a CD8+ T cell deactivating per tick. |
| *ActivatedCD8TNFSecretion* | Molecules of TNF-α secreted from an activated CD8+ T cell in one second. |
| *ActivatedCD8IFNSecretion* | Molecules of IFN-γ secreted from an activated CD8+ T cell in one second. |
| *cd8PopulationDoublingTime* | The length of time in hours it takes an activated CD8+ T cell to proliferate. |
| *maximumCD8Generations* | The maximum number of times a single CD8+ T cell can proliferate. |
| *CD8KillProbability* | The probability a CD8+ T cell will kill an infected macrophage and the intracellular bacteria per tick given the CD8+ positive T cell has an infected macrophage in its Moore neighborhood. |
| **Diffusion** |  |
| *TNFthresholdForImmuneCellMovement* | Number of TNF-α molecules in the local Moore neighborhood including middle grid cube of the current cell required for probabilistic movement rather than random movement. |
| *TNFDiffusionCoefficient* | The diffusion coefficient (10^-7 cm^2/s) used to simulate TNF-α diffusion. |
| *TNFDegradationRatePerSecond* | Rate of TNF-α degradation in 1/s. |
| *IFNDiffusionCoefficient* | The diffusion coefficient (10^-7 cm^2/s) used to simulate IFN-γ diffusion. |
| *IFNDegradationRatePerSecond* | Rate of IFN-γ degradation in 1/s. |
| *granulomaFractionOfDiffusion* | The fraction that diffusion is slowed within granulomas. |
| *sphereEfficiency* | The fraction of grid cubes in the sphere that are initialized to contain cells. |
