# Supplementary material for: Agent-based model predicts that layered structure and 3D movement work synergistically to reduce bacterial load in 3D in vitro models of tuberculosis granuloma
Source: PLoS Comput Biol. 2024 Jul 12;20(7):e1012266. doi: 10.1371/journal.pcbi.1012266 (PMC11288457; doi:10.1371/journal.pcbi.1012266)
Supplement: S2 Table — (DOCX) [file pcbi.1012266.s002.docx]

**S2** **Table.** Parameters that were held constant during sampling, their values, and units.

| **Parameter** | **Constant value** | **Units** |
| --- | --- | --- |
| **Traditional v Spheroid** | | |
| CaseNumber | Spheroid: 9  Traditional: 15 | - |
| isSpheroid | Spheroid: 1  Traditional: 0 | - |
| gridDim_X_Y | Spheroid: 80  Traditional: 216 | Grid squares |
| gridDim_Z | Spheroid: 80  Traditional: 11 | Grid squares |
| **Simulation defined** | | |
| isBatchRun | 1 | - |
| cellsNeededToBeAddedToGran | 8 | Neighboring immune Cells |
| isPlainColors | 6 | - |
| granQualificationImmuneCellCount | 27 | Immune cells |
| randomSeed | Random | - |
| DiffusionTimeStepMultiplier | 4 | - |
| timestep | 6 | Minutes/step |
| agentLimit | 60000000 | Agents |
| cellsNeededToRemainInGran | 8 | Neighboring immune cells |
| divisionBiomassThreshold | 2 | - |
| **Experimentally defined** | | |
| daysToRun | 6 | Days |
| fractionCD3 | 1 | CD3+ cells/lymphocytes |
| InitialPBMCs | 100000 | Cells |
| fractionMonocyte | 0.4 | Monocyte/PBMC |
| fractionLymphocyte | 0.6 | Lympocyte/PBMC |
| timeToAddTcells | 48 | Hours |
| **Variance** | | |
| cd4DoublingTimeVariance  cd8DoublingTimeVariance | 0.25 |  |
| nfkbVariance | 0.1 |  |
| mtbGrowthRateVariance | 0.1 |  |
| stat1Variance | 0.1 |  |
| macrophageLifeSpanVariance | 0.1 |  |
| cd4LifeSpanVariance  cd8LifeSpanVariance | 0.1 |  |
| divisionBiomassVariance | 0.2 |  |
| newMtbPlacementRange | 0.2 |  |
| MinBurstLimit | 20 | Internal bacteria (1) |
| MaxBurstLimit | 40 | Internal bacteria (1) |

References

1. Repasy T, Lee J, Marino S, Martinez N, Kirschner DE, Hendricks G, et al. Intracellular Bacillary Burden Reflects a Burst Size for Mycobacterium tuberculosis In Vivo. Behr MA, editor. PLoS Pathog [Internet]. 2013 Feb 21 [cited 2021 May 24];9(2):e1003190. Available from: https://pubmed.ncbi.nlm.nih.gov/23436998/
