# Supplementary figures and images for: Agent-based model predicts that layered structure and 3D movement work synergistically to reduce bacterial load in 3D in vitro models of tuberculosis granuloma
Source: PLoS Comput Biol. 2024 Jul 12;20(7):e1012266. doi: 10.1371/journal.pcbi.1012266 (PMC11288457; doi:10.1371/journal.pcbi.1012266)

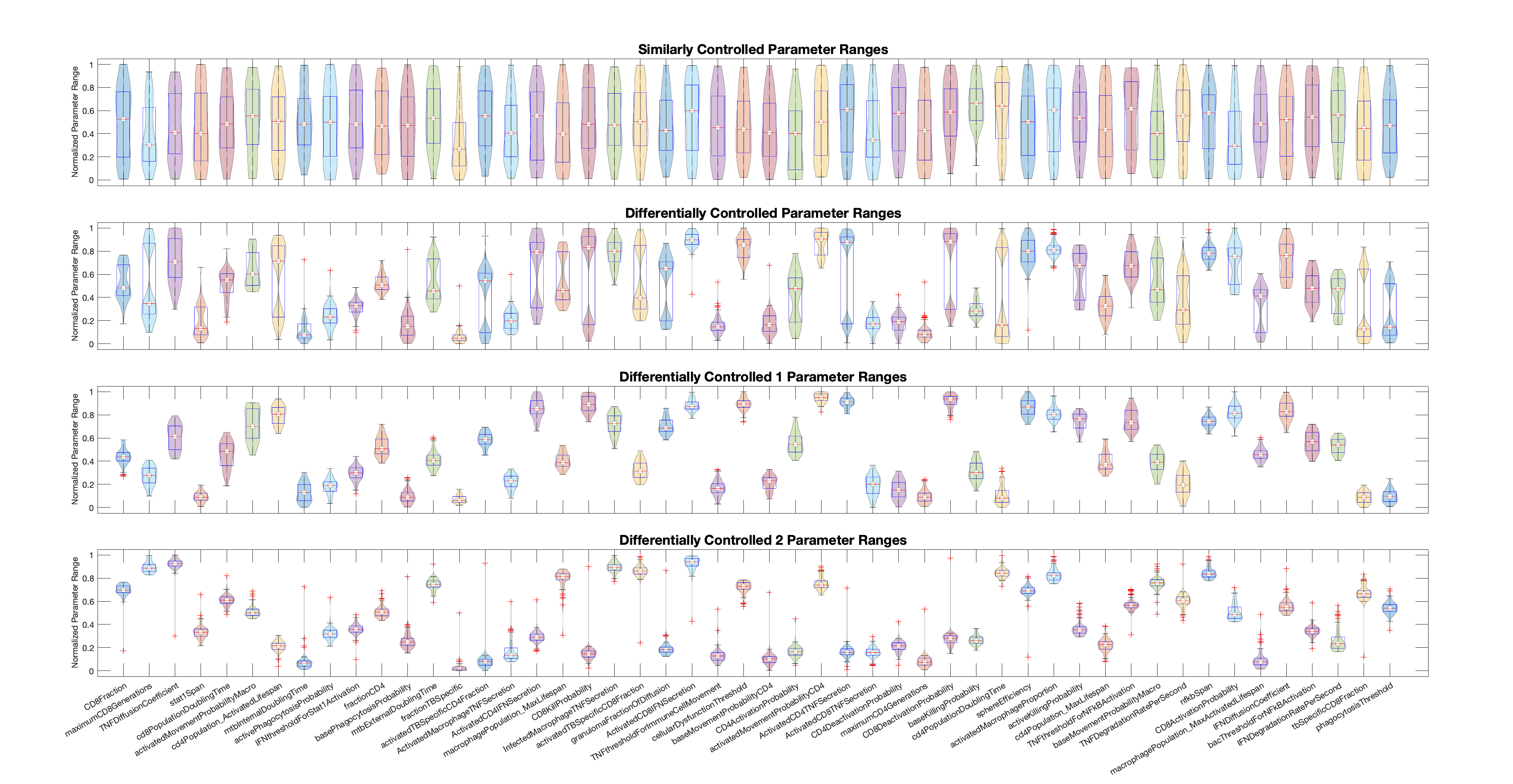

Supplement: S1 Fig — Each parameter’s range has been normalized from 0 to 1 based on the initial lower and upper bounds of the parameters ranges as given in Table 1. (TIF) [file pcbi.1012266.s003.tif]

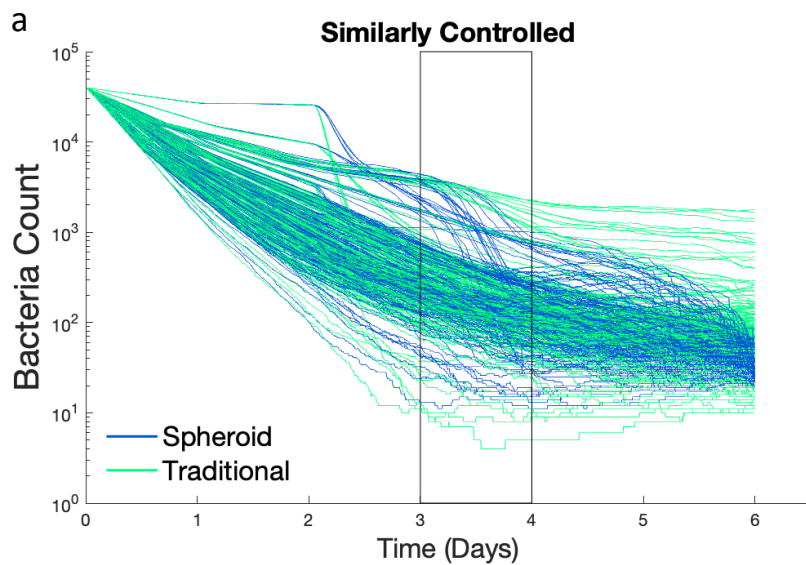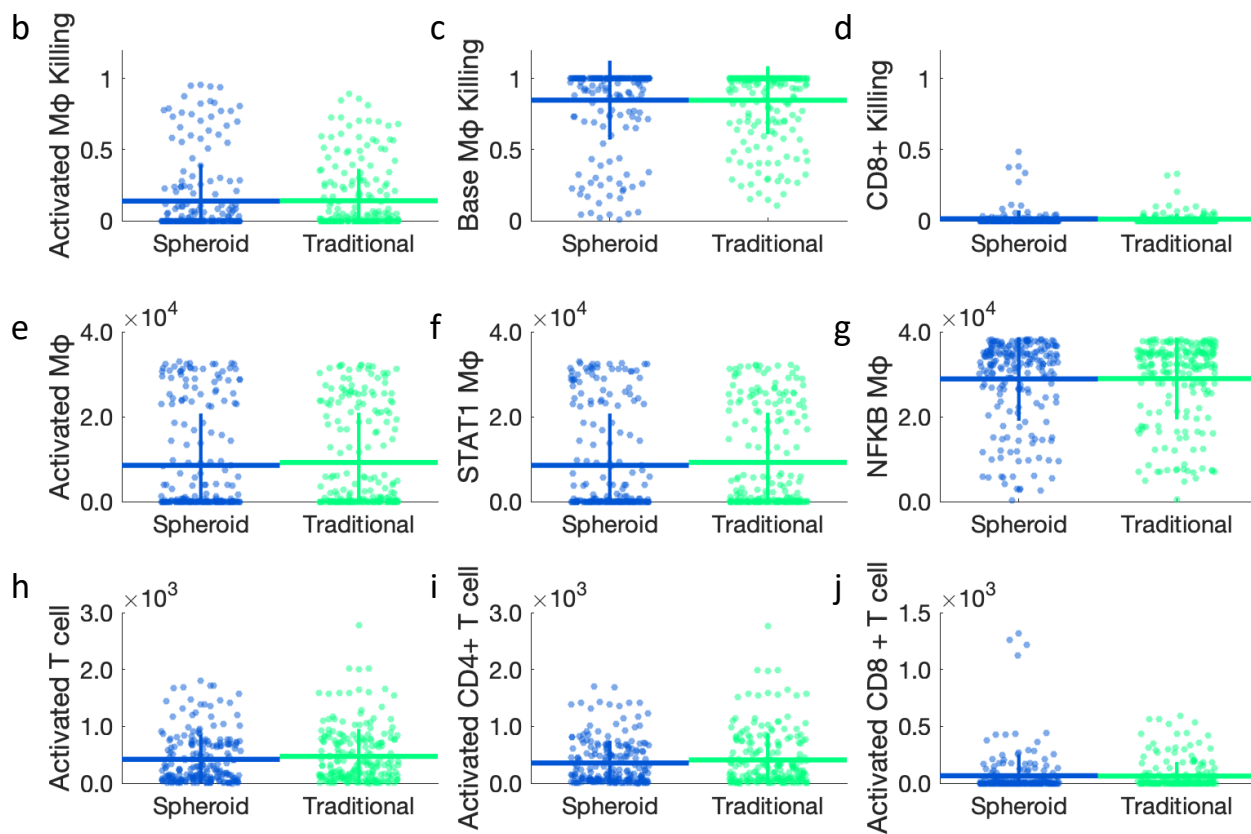

Supplement: S2 Fig — a) Bacterial dynamics for similarly controlled simulations. The time range of interest (Day 3–4) is outlined. These are the same time courses as shown in Fig 3b. Proportions of bacterial killing due to b) activated macrophages, c) base macrophages, and d) CD8+ T cell killing from day 3 to 4. Comparing total counts of e) activated macrophages, f) STAT1 activated macrophages, g) NF-κB activated macrophages, h) activated T cells, i) activated CD4+ T cells, and j) activated CD8+ T cells between spheroid and traditional simulations at day 4. (PDF) [file pcbi.1012266.s004.pdf]

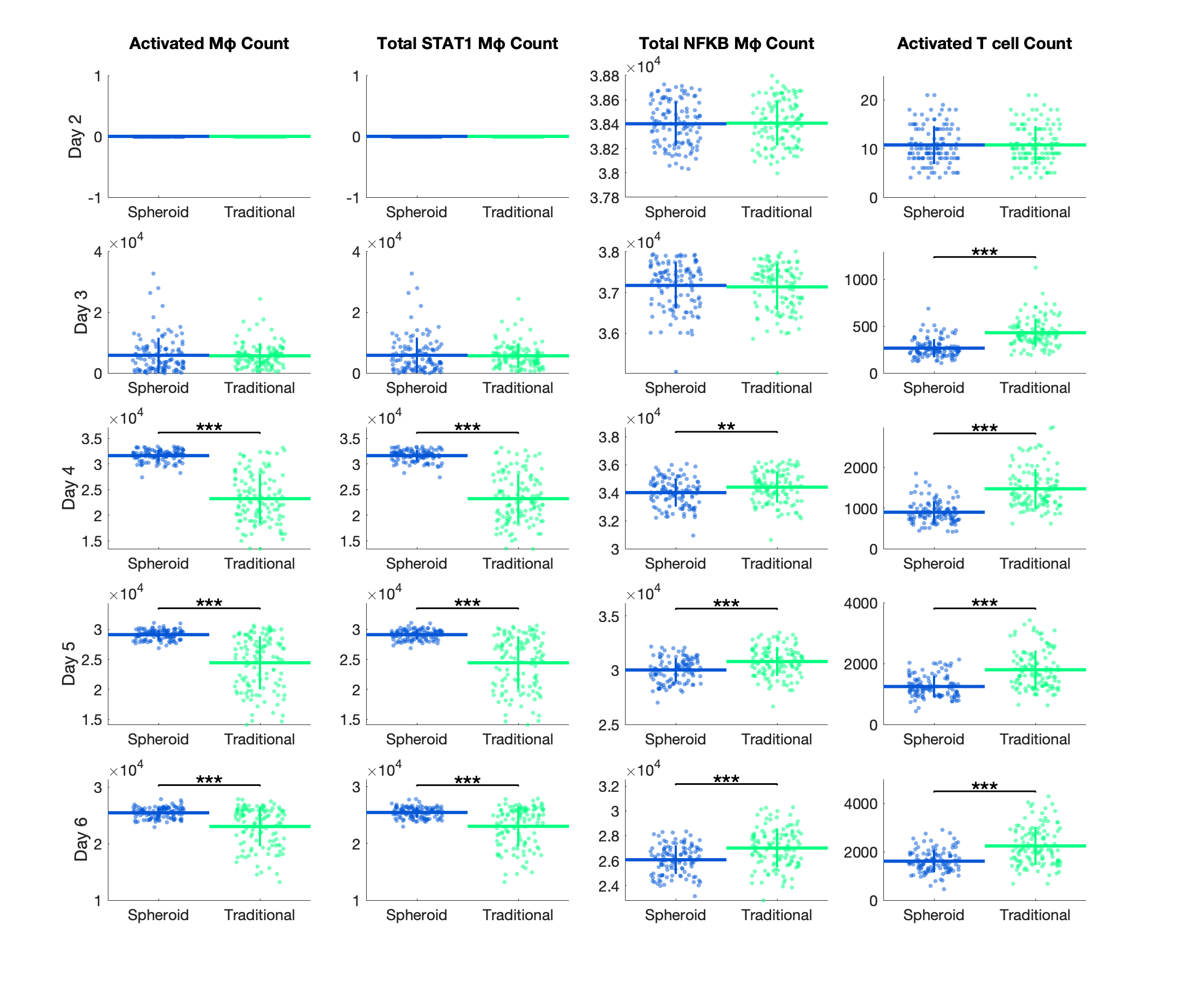

Supplement: S3 Fig — (TIF) [file pcbi.1012266.s005.tif]

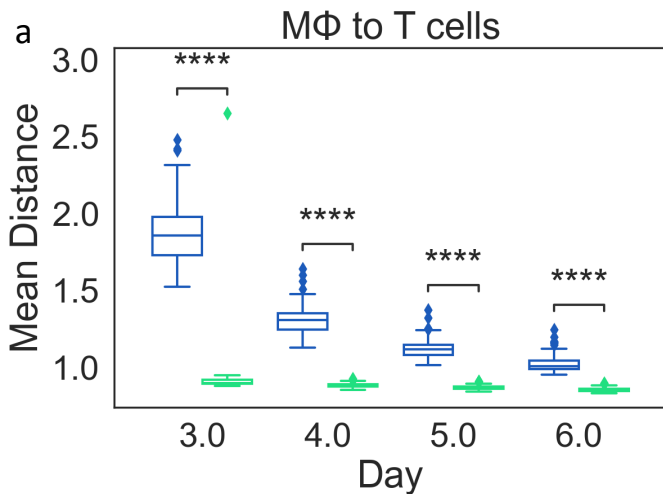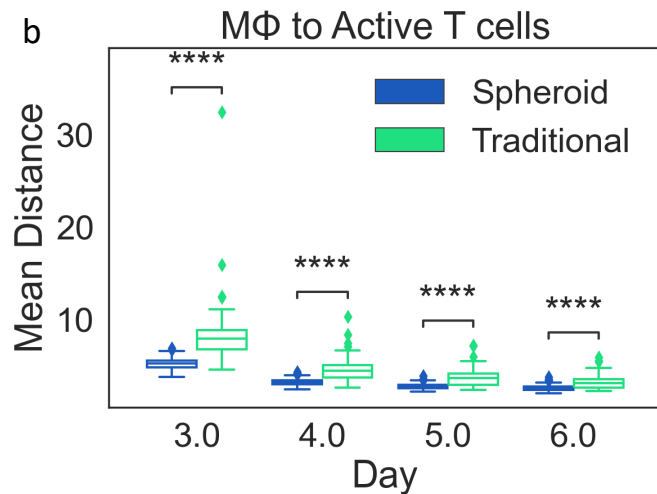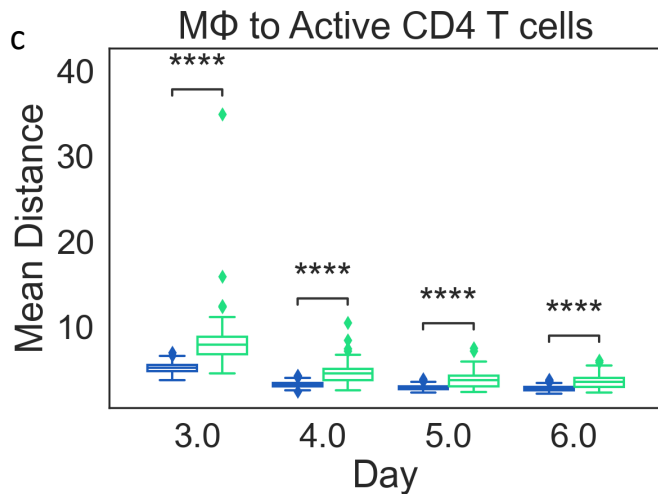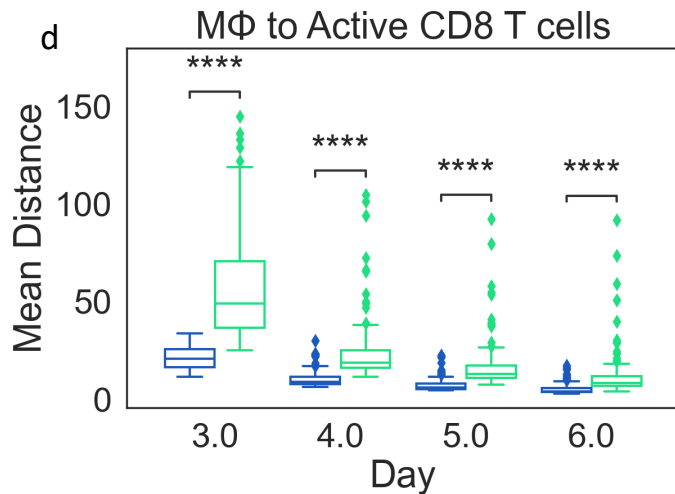

Supplement: S4 Fig — The mean distance from macrophages to nearest a) T cells, b) activated T cells, c) activated CD4+ T cells, and activated CD8+ T cells. Each data point represents an average of all cells in a single simulation. The distribution of the mean distances across the set of simulations is compared between DC-1 spheroid and traditional simulations from day 3 to day 6. (PDF) [file pcbi.1012266.s006.pdf]

**Activated CD8+ T cell Count**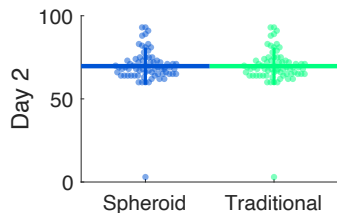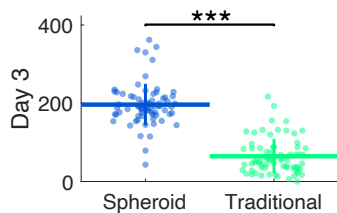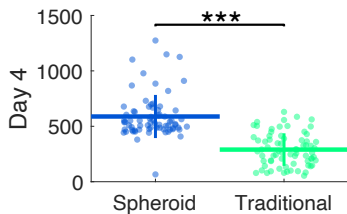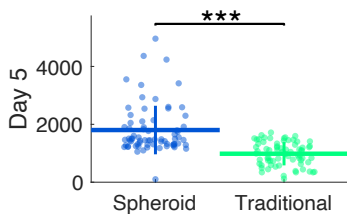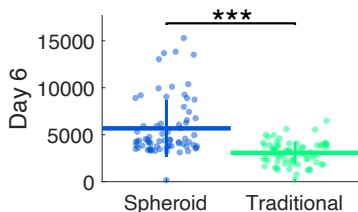**Total STAT1 M $\phi$  Count**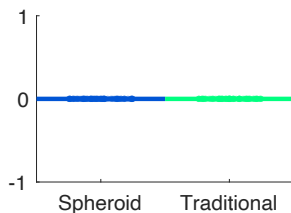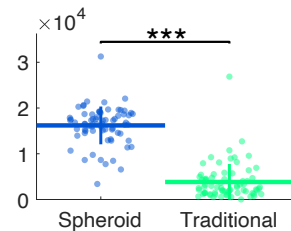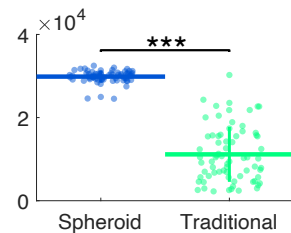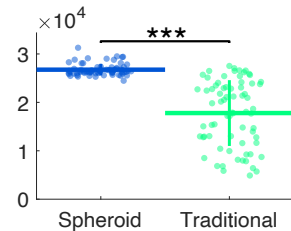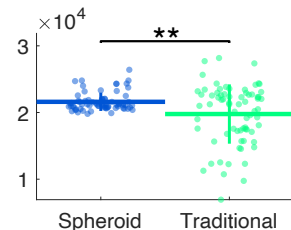**M $\phi$  Interacted with Bac Count**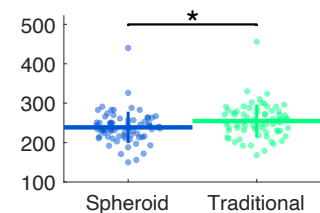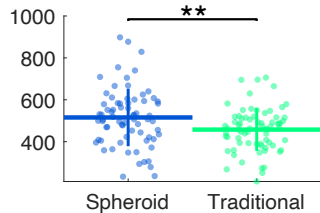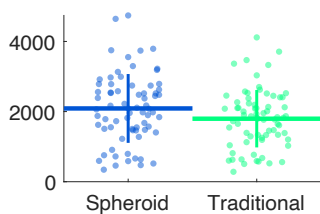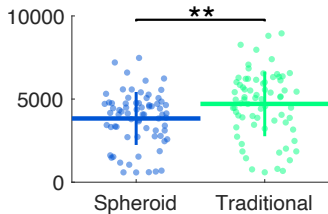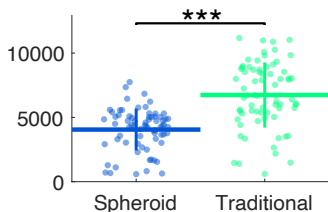

Supplement: S5 Fig — * p< = 1e-1, ** p< = 1e-2, *** p< = 1e-3. (PDF) [file pcbi.1012266.s007.pdf]

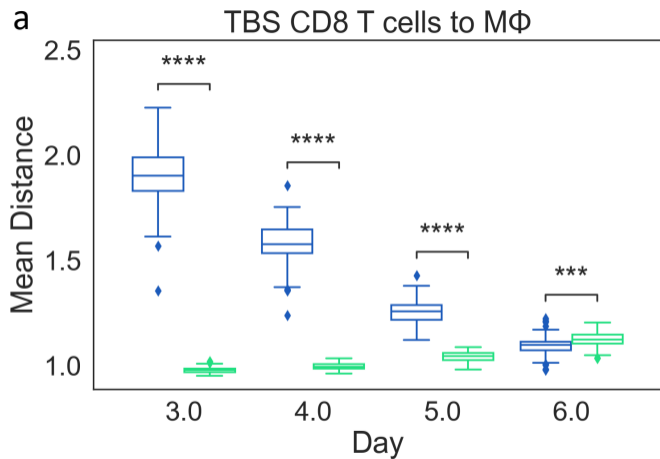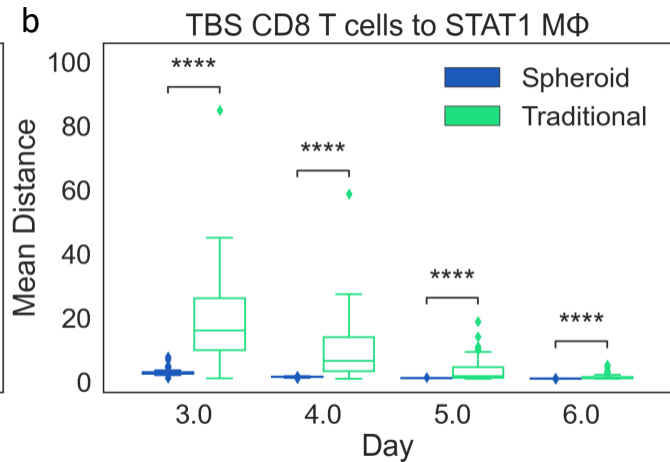

Supplement: S6 Fig — The mean distance from a) TB-specific CD8+ T cells to nearest macrophage and b) TB-specific CD8+ T cells to nearest STAT1 activated macrophage. Each data point represents an average of all cells in a single simulation. The distribution of the mean distances across the set of simulations is compared between DC-2 spheroid and traditional simulations from day 3 to day 6. * p< = 0.05, *** p< = 1e-3, **** p< = 1e-4. (PDF) [file pcbi.1012266.s008.pdf]

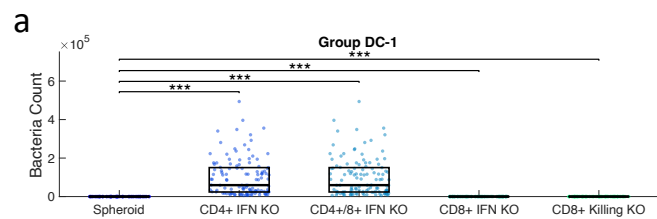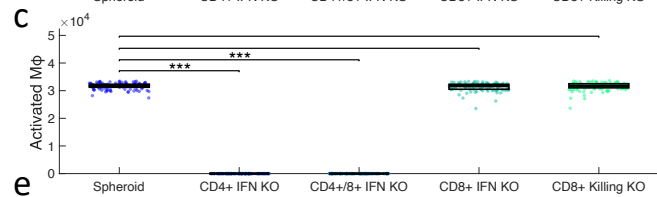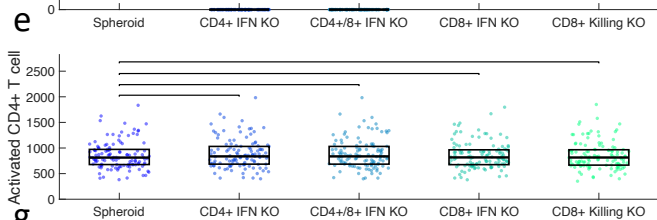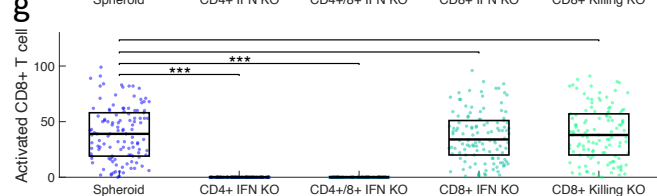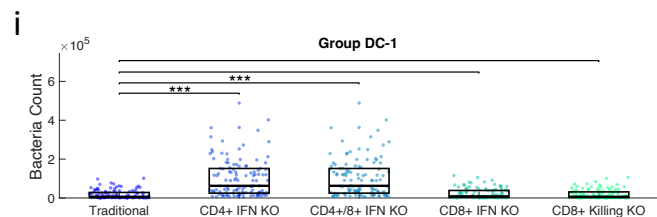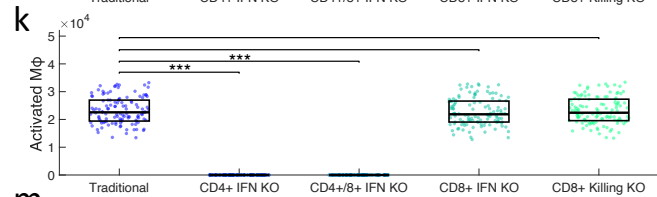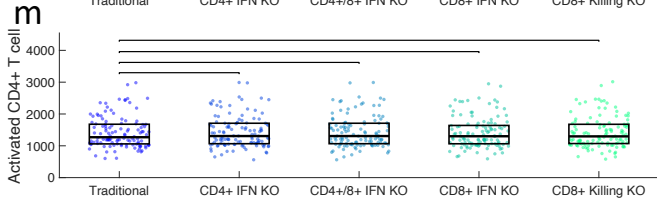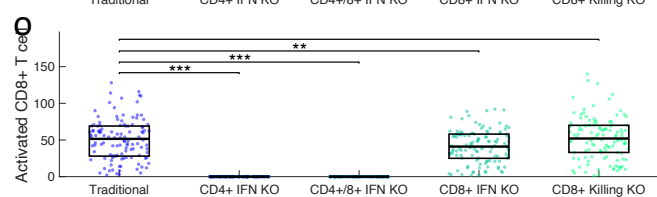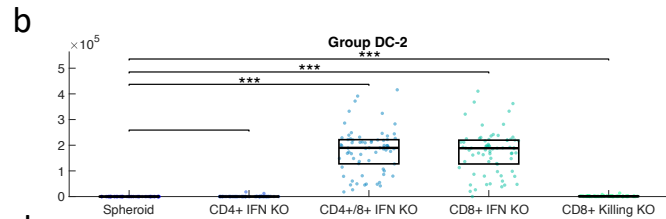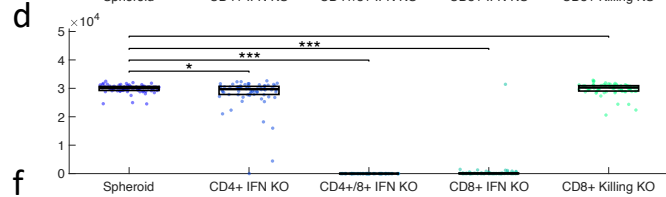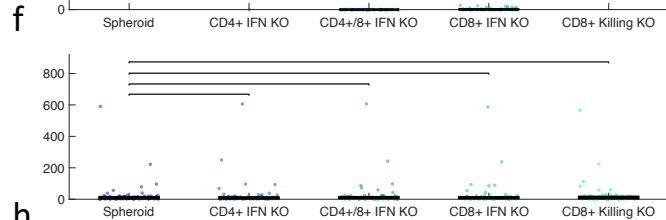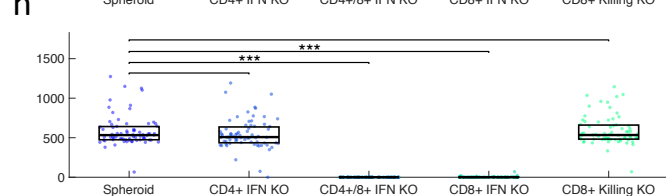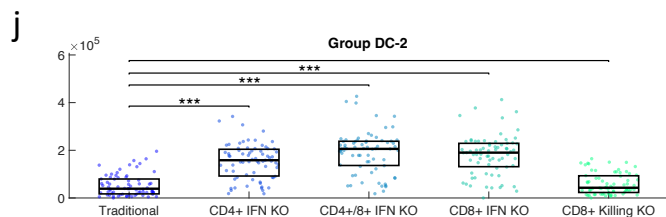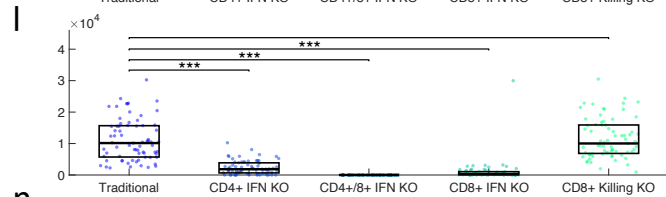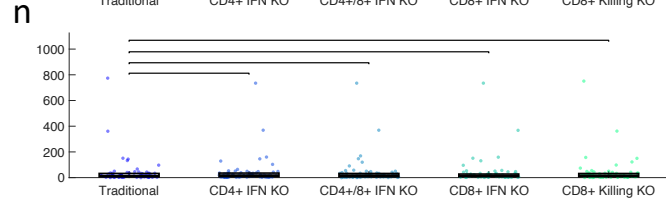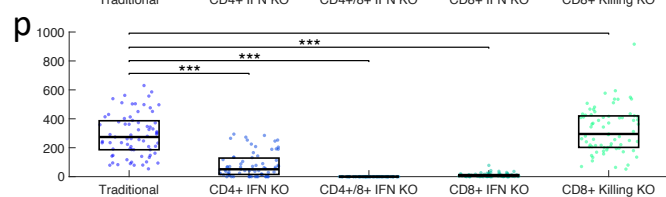

Supplement: S7 Fig — A simulation knocking out CD8+ T cell killing of infected macrophages was also performed. The original simulation and the 4 KO scenarios were compared by looking at ab,I,j) bacterial counts at day 6, c,d,k,l) activated macrophage counts at day 4, e,f,m,n) activated CD4+ T cells counts at day 4, and g,h,o,p) activated CD8+ T cell counts at day 4. These outputs are shown for the DC-1 spheroid simulations (a,c,e,g), DC-2 spheroid simulations (b,d,f,h), DC-1 traditional simulations (I,k,m,o), and DC-2 traditional simulations (j,l,n,p). (PDF) [file pcbi.1012266.s009.pdf]

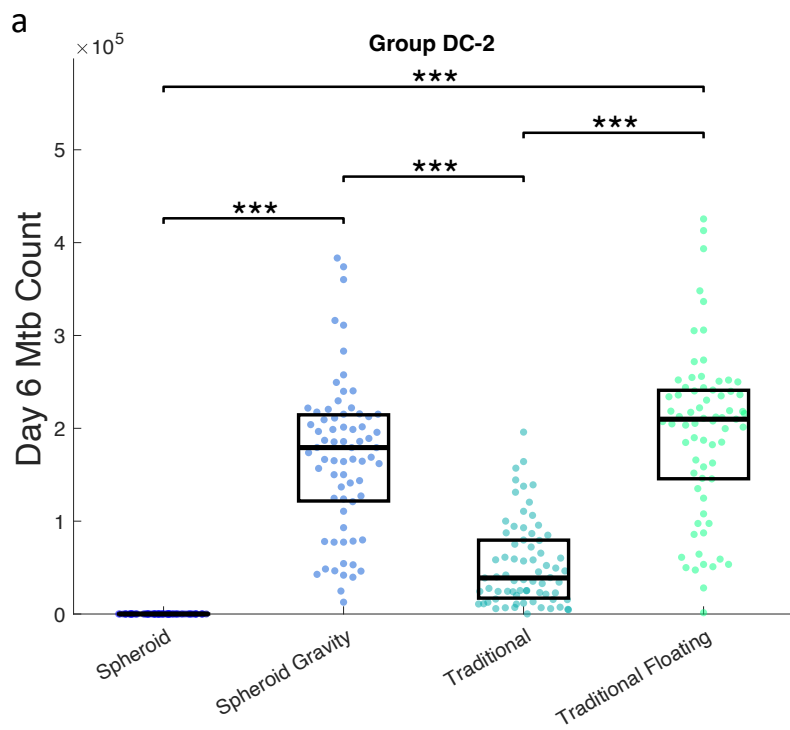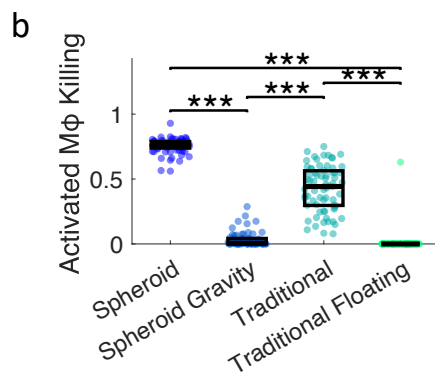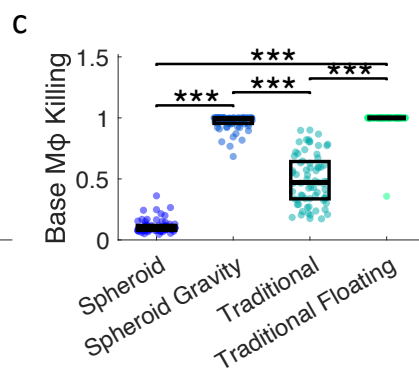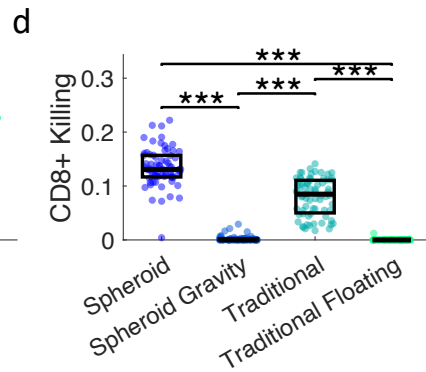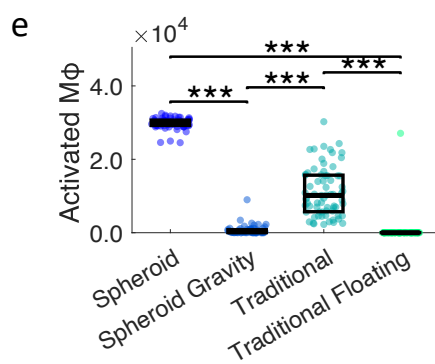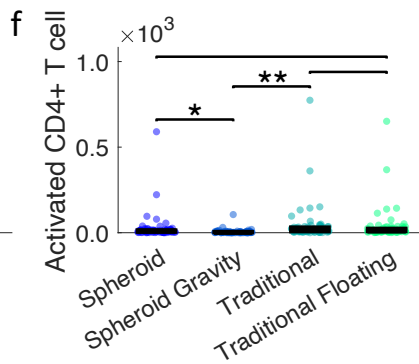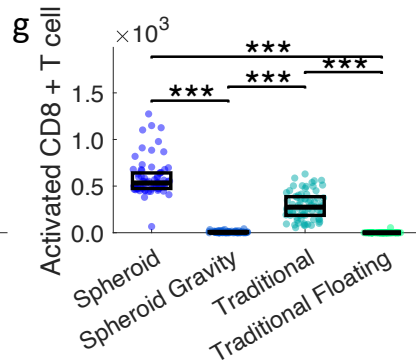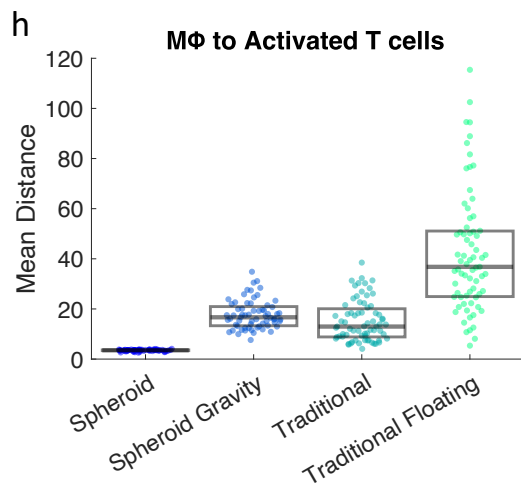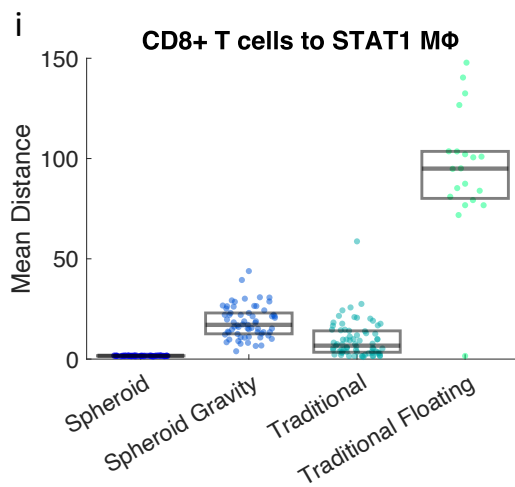

Supplement: S8 Fig — Comparisons between the original setups (spheroid and traditional) and those with swapped movement rules (spheroid gravity and traditional floating) for Mtb count at day 6(a); proportion of bacterial killing due to activated macrophages (b), base macrophages(c), and CD8+ T cell cytotoxic killing of macrophages and internalized bacteria(d) from day 3–4; and levels of activation in macrophages(e), CD4+ T cells(f), and CD8+ T cells(g). * p< = 0.05, ** p< = 1e-2, *** p< = 1e-3. (PDF) [file pcbi.1012266.s010.pdf]

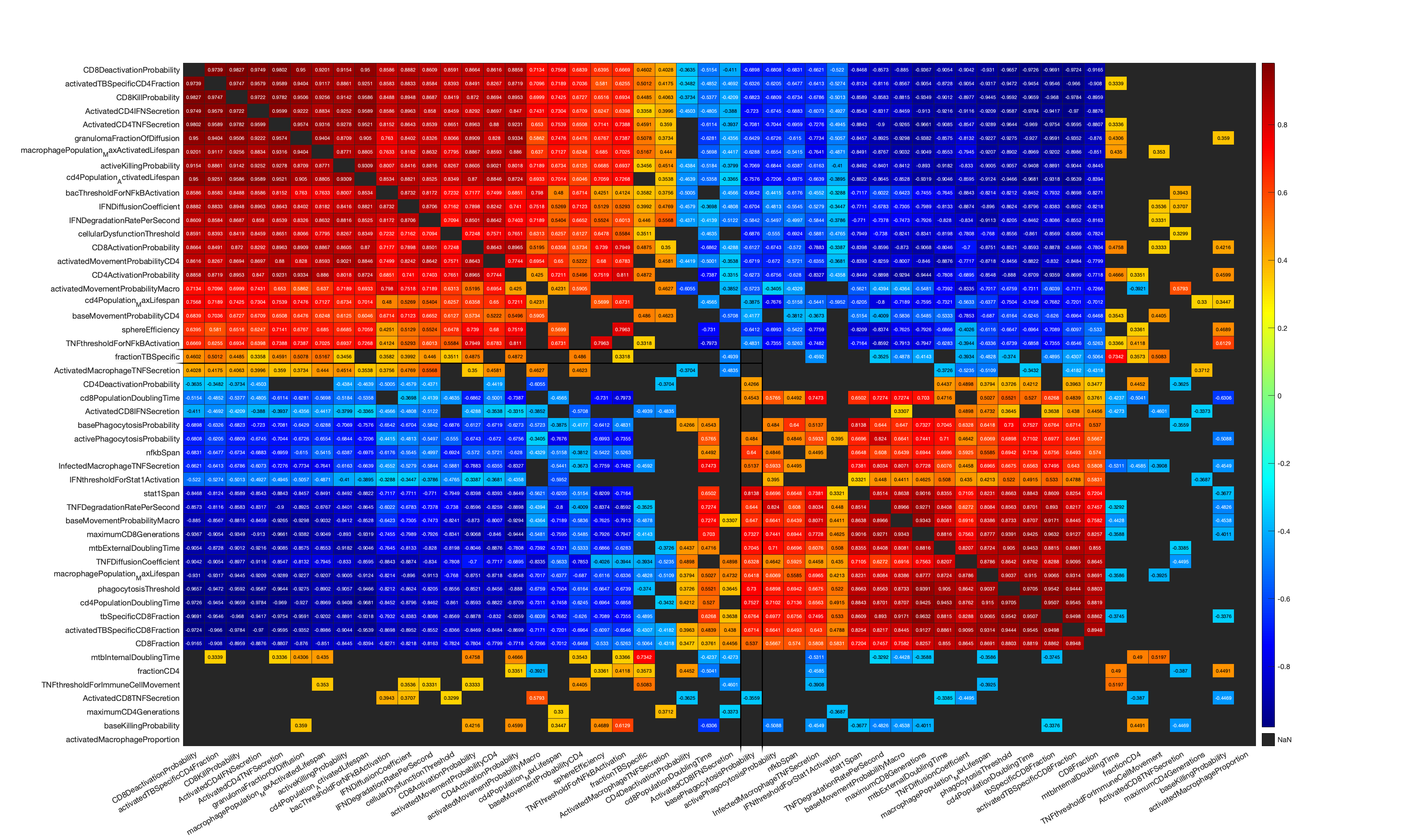

Supplement: S9 Fig — (p = 0.01 with Bonferroni correction). (TIF) [file pcbi.1012266.s011.tif]
