# Supplementary material for: Agent-based model predicts that layered structure and 3D movement work synergistically to reduce bacterial load in 3D in vitro models of tuberculosis granuloma
Source: PLoS Comput Biol. 2024 Jul 12;20(7):e1012266. doi: 10.1371/journal.pcbi.1012266 (PMC11288457; doi:10.1371/journal.pcbi.1012266)
Supplement: Attachment — Submitted filename: Elsjefinal.docx [file pcbi.1012266.s012.docx]

Agent-based model predicts that layered structure and 3D movement work synergistically to reduce bacterial load in 3D in vitro models of tuberculosis granuloma

Submitting to: PLOS Computational Biology

- This is an exciting paper combining both wetlab and modeling studies to explore open questions in the immune response to Mtb infection. Overall the paper is well-written, and lays out nicely the differences between different in vitro approaches and how modeling was used to dissect those distinctions and the outcomes.  A few minor points and general comments.

- --More detail about how the results and predictions of the modeling analysis and how it was performed and the approach(es) used in Figures 5-7 is needed.

- Line 71 you say: layout of specific cells better to say spatial organization

- Lines 80-95  can you elaborate what gets put into the “dishes” for the 2-D or 3-D to represent the immune response (cytokines? Cells? Which ones? A quick list to give an idea would help To distinguish from the biomimetic that follows.

- Line 101 needs a reference.

- Line 105 fix ref 13 before period. (in many cases in text reference is after period).

- Line 113—this has been observed in PMCID: [7855029](http://www.ncbi.nlm.nih.gov/pmc/articles/PMC7855029), (I see you reference it at line 675)

- Lines 80-109 YOU Need to refer to Figure 1! Would have addressed point above

- Line: 212—‘knockout’ is a colloquial term that doesn't explain what it is ---.suggest that you use the phrase “ virtual gene deletion (i.e. virtual knockout)” to clarify to audience first time – also be sure that it is clear if it is the model in each case that is knocking out or the in vitro system.

- Line: 223- figure 2 legend should we assume there is no 2-D simulation? Since you only mention 3-D? maybe clarify?

- Line: 241 legend 3—I missed where/how the  in vitro dataset shown was  generated/described? Needs methods or clarity. (also line 258 “experimental data at day 6.”

- Line:263: data ARE

- Line 264 this second part is not clear : “a wide data range to calibrate to at the end time point.” ??

- Line 350—could you remove the ability of activated macs to be activated to see if that attenuates?

- Line: 514- I was thinking a results table that outlines the different conditions and findings would be cool and helpful.

- Line 586: the authors acknowledge there is large potential for parameter uncertainty but do not do an uncertainty or sensitivity analysis in this work which is odd..can they clarify? A  sensitivity analysis would certainly formalize many of the results that they predicted and would tandem nicely on the correlations between wet and computer.

- Also the authors refer to “appendix” throughout. Do they mean “supplement”?
- This is an exciting paper combining both wetlab and modeling studies to explore open questions in the immune response to Mtb infection. Overall the paper is well-written, and lays out nicely the differences between different in vitro approaches and how modeling was used to dissect those distinctions and the outcomes.  A few minor points and general comments.

- The paper is well-written for the most part, but a few minor comments:
  - There are several times when it seems like “shorthand” or “speech-like” verbiage is used.  For example, line 27: “Higher T cell activation” should probably be “Higher T cell activation rate”. In line 293, you have “The bimodal distributions **do** correspond…” as opposed to “The bimodal distributions correspond…”
  - There seems to be a mix of active/first person tense (e.g. line 257-265) and passive/third person tense (e.g. line 266-279).
  - Line 71 you say “layout of specific cells”, better to say “spatial organization”
  - Line 105 fix ref 13 before period. (in many cases in text reference is after period).
  - Line 162: “Bacteria grow…” is in present tense, but Line 165-166 has “Macrophages will…” which is in future/conditional tense.
  - Line:263: data ARE
  - Line 264 this second part is not clear : “a wide data range to calibrate to at the end time point.” ??
  - Line 265: “heterogeneous” is redundant.
  - Line 350—could you remove the ability of activated macs to be activated to see if that attenuates?
  - The figures don’t gray-scale very well for print - the light-green color for “traditional” are very, very light. Perhaps red or a darker green would be an easy change?
  - The y-axis bars in figure 8 are inconsistently styled between rows 1 and 2.
  - Check grammar/revise wording on line 577; it was both an important statement and somewhat difficult to parse.
  - Line 92 - General modelers may not know what PBMCs are *a priori*.
- More detail about how the results and predictions of the modeling analysis and how it was performed and the approach(es) used in Figures 5-7 is needed.
- Lines 80-95  can you elaborate what gets put into the “dishes” for the 2-D or 3-D to represent the immune response (cytokines? Cells? Which ones?) A quick list to give an idea would help to distinguish from the biomimetic that follows.
- Line 101 needs a reference.
- Line 113—this has been observed in PMCID: [7855029](http://www.ncbi.nlm.nih.gov/pmc/articles/PMC7855029), (I see you reference it at line 675)

- Lines 80-109 YOU Need to refer to Figure 1! Would have addressed point above
- Are parameters uniformly sampled? A table of parameters, ranges, distributions, and references for how those distributions were selected would help keep the reader grounded throughout the paper.
- Line: 212—‘knockout’ is a colloquial term that doesn't explain what it is ---.suggest that you use the phrase “ virtual gene deletion (i.e. virtual knockout)” to clarify to audience first time – also be sure that it is clear if it is the model in each case that is knocking out or the in vitro system.
- Line: 223- figure 2 legend should we assume there is no 2-D simulation? Since you only mention 3-D? maybe clarify?
- It is sometimes difficult to tell whether certain italicized quantities are (input) parameters, intermediate behaviors, or outcomes. For example, I don’t know if *CD8KillProbability* is an independent model input or if this is an emergent quantity internal to the model based on other factors. This is doubly important because for some (*cd4doublingtime*) appears to be the main bifurcation parameter about which the DC-1/DC-2 delineation are based.
- There was a statement that “Several parameters [show] bimodal distributions” which appears to be a very substantial claim.  This suggests that there’s another mechanism, not represented, that switches between two modes - and whatever those modes are seem to be a major differentiator between DC-1 and DC-2. I find myself wondering “What is the biological interpretation of that bimodal distribution? What is that other mechanism?” Can you comment more about the biological intuition/hypothesis that drives your distinction between DC-1 and DC-2?
  - Moreover, it seems like there should be some sort of sensitivity analysis performed to determine the impact of certain parameters (e.g. *cd4doublingtime*) on bacteria count as further quantitative support for the use of that parameter to split the runs into DC1/DC2.  If this is not a reasonable ask, then perhaps you can comment on the computational burden of running these simulations (e.g. if they require a lot of GPU usage, wall time, etc.) Or, perhaps make a clearer statement that a one-at-a-time SA was run - perhaps something that looks like Figure 3, but where you only vary one parameter.
- I am curious to know whether reducing activated macrophage killing in spheroid simulations causes them to return to traditional-like killing rates.
- In Figure 8, it seems like there’s about a 100x or more difference between traditional and spheroid CFU counts in the mock/control condition. Does this comment on the quality of traditional *in vitro* culturing? Do you expect the relative fold-changes between spheroid-control and spheroid-knockout to be similar to the fold-changes in the traditional analogue?
- In paragraph starting line 509, the authors seem to make a substantial claim about the relative roles of CD8+ and CD4+ T cells, specifically that “...a CD4+ T cell dominant response in DC-1 simulations…” However, the DC1/DC2 conditions appear to be defined based on the ability of CD4+ T cells to proliferate. Does this suggest some sort of “compensatory role” that you expect CD8+ T cells to be playing?
- In section 3.7, the authors claim that “traditional floating” doesn’t fare as well as traditional in some ways. In “traditional floating”, was there a control for any sort of “mean horizontal speed” of cells? Do additional degrees of freedom “slow them down”? I would be interested in the author’s insights as to why traditional-floating had such a different outcome.

- Line: 514- I was thinking a results table that outlines the different conditions and findings would be cool and helpful.
- For the spheroid-gravity simulations that controlled very poorly, were the simulation initial conditions similar to the spheroid in terms of the spatial distribution of T-cells and macrophages? I am left to speculate that the T cells are being “Gravity filtered” out of the simulated *in vitro* granuloma, like in a column filter.
- Line 586: the authors acknowledge there is large potential for parameter uncertainty but do not do an uncertainty or sensitivity analysis in this work which is odd..can they clarify? A  sensitivity analysis would certainly formalize many of the results that they predicted and would tandem nicely on the correlations between wet and computer.

- Also the authors refer to “appendix” throughout. Do they mean “supplement”?
