# Supplementary material for: Agent-based model predicts that layered structure and 3D movement work synergistically to reduce bacterial load in 3D in vitro models of tuberculosis granuloma
Source: PLoS Comput Biol. 2024 Jul 12;20(7):e1012266. doi: 10.1371/journal.pcbi.1012266 (PMC11288457; doi:10.1371/journal.pcbi.1012266)
Supplement: Attachment — Submitted filename: 2024_04_07_PLOSCB_RtR.docx [file pcbi.1012266.s013.docx]

**Editors:**

Overall, the reviewers indicate a need for clarification of the modeling approach, the justifications for this approach, and the results.

In particular, although the full model is explained in a separate preprint and the parameter ranges are tabled in the supplement, the reviews indicate that it would benefit this manuscript to include a brief description of the parameterization process and of the parameter distributions/constraints in the main text.

We have added a section to clarify the calibration process. The simulations analyzed are composed of a final set of parameters sets that fall within experimental ranges of data. Distributions are shown in S1 Fig.

Further, it should be made very clear early in the manuscript that this model is hypothesis generating, and the results should be presented more explicitly in that context.

This is a great suggestion. We have added this statement in the introduction and discussion, and made edits throughout the paper to more clearly present our work in this context.

**Reviewer #1:**

The manuscript 'Agent-based model predicts that layered structure and 3D movement work synergistically to reduce bacterial load in 3D in vitro models of tuberculosis granuloma' by Petrucciani et al. aims at using two different topologies of an agent based model of tuberculosis granuloma. One topology corresponds to a 3D in-vitro model where cells can move freely in three directions and that is initialized with a core of macrophages and a shell of T cells, whereas the other corresponds to a 2D layer in-vitro model. The simulations contain macrophages, CD4 and CD8 T cells and bacteria.
Cells can secrete cytokines and move along chemokine gradients. Direct and cytokine mediated cell-cell contacts can change the activation status of cells.
The authors find different types of behavior in 3D and 2D simulations and discuss possible reasons.

The authors refer to the mechanisms described in their model by describing them as 'established' rules that have been previously published. 'This hybrid model was
parameterized in Petrucciani et al. with experimental data for bacterial fold change, cell count, and cell viability at day 6 from both the spheroid and the traditional cultures.' Since the cited publication has not been peer reviewed, it is unclear in what sense it represents an 'established' model.

Since the original submission, this prior manuscript has been peer reviewed and published at PLOS ONE (15). (https://doi.org/10.1371/journal.pone.0299107)

Even though the manuscript does not describe the parameter variation process explicitly, Table 1 and its caption and some discussions in the manuscript suggest that parameters ranges are varied and values are drawn from certain distributions, with the structure of the latter remaining unclear.

We have added a section to clarify the calibration process which would hopefully help address this concern also. The simulations analyzed are composed of a final set of parameter sets that produce results that fall within experimental ranges of data. The final distributions of the calibrated parameters are shown in S1 Fig.

In fact, many mechanisms and parameters remain unclear. For instance, what determines chemokine secretion? How are chemotactic gradients established? How do they depend on cellular states?

We originally kept the model structure brief because additional detail can be found in the preceding paper. Based on your suggestion we have extended the methods to include further detail on chemokines secretion, diffusion, and chemotaxis.

The aim of the manuscript is very laudable. Computational models offer the opportunity to analyze which mechanisms are responsible for certain features in experimental data. However, care must be taken to constrain model parameter values as tightly as possible based on experimental data. Here, the constraint is just one time point with cell and bacteria counts. For the amount of parameters that are varied, this is clearly insufficient, rendering the detailed discussion of the reasons behind the observed differential behavior of the model topologies rather moot, in my view.

We agree that ideally computational models should be calibrated to as much data as possible, and that our current dataset is limited. However, our model is also informed by and constrained by immunological mechanisms and parameter ranges from literature. We worked with what system-specific data was available and calibrated to try to fill the experimental range of bacterial counts allowing many different bacterial dynamics. Further we present and analyze multiple varying dynamics that were seen within the calibrated set, thereby accounting for model uncertainty in our interpretations.

We believe this modeling work is worthwhile because it can 1) help pick important data points and time points that should be measured in the future and 2) can assist in generating hypotheses that explain experimentally observed differences. Together, this encourages more experimental data to be generated, makes the existing data more informative for the model calibration, and improves future iterations of the model. We have updated the manuscript text to reframe this model as hypothesis generating to help clarify these limitations.

**Reviewer #2:**

This paper presents and explores the dynamics of an agent-based in silico TB infection model. The paper was well-organised and clearly written. Experimental data was incorporated to parameterise the model and used to compare against model behaviour. Results were clearly presented using appropriate statistics and the discussions of results were well-reasoned. The results and conclusion reached by the paper could have significant impact on furthering our understand of TB.

The references section needs careful proof reading and editing as it contains numerous errors: incomplete references (e.g. (5), (24)) and formatting errors (e.g. (10); (13); (22,23) - journal name inconsistent; (12) - full journal name).

Thank you for identifying these inconsistencies. We used Mendeley software for our citations and have gone back to check the Mendeley information used to generate citations. We have also checked the generated citations for completeness.

Figure 1 p.9 - The caption of this figure needs more detail. In (B) the desciption doesn't describe the figure (e.g. CD3/PBMC are not mentioned in the text). Also, there's a mismatch between "B" and "b" (inconsistent use of upper/lower case).

We have corrected the case inconsistence and added more detail to the Figure 1 caption.

There were a number of minor typographic errors which did not affect understanding but need correction:

* p.6 "An additional 10.6 people fell ill" - 10.6 people?

This has been corrected to 10.6 million.

* throughout, remove contractions e.g. p.6 "hasn't"; p.25 "don't", p.41 "don't".

We have removed contractions throughout the manuscript.

* throughout, references needs to be before full stops. e.g. p.6 "decades.(1)" -> "decades (1)."; The same comment applies to Figure and Appendix references.

Citation, figure, and appendix references have been moved before full stops.

* throughout, inconsistent use of bold font in Figure/Section references e.g. p.10 "Figure 1a" ("Figure 1" is in bold, "a" is not).

We have un-bolded all Figure references for consistency.

Reviewer #3:

Agent-based model predicts that layered structure and 3D movement work synergistically to reduce bacterial load in 3D in vitro models of tuberculosis granuloma

Submitting to: PLOS Computational Biology

- This is an exciting paper combining both wetlab and modeling studies to explore open questions in the immune response to Mtb infection. Overall the paper is well-written, and lays out nicely the differences between different in vitro approaches and how modeling was used to dissect those distinctions and the outcomes.  A few minor points and general comments.
- The paper is well-written for the most part, but a few minor comments:
  - There are several times when it seems like “shorthand” or “speech-like” verbiage is used.  For example, line 27: “Higher T cell activation” should probably be “Higher T cell activation rate”. In line 293, you have “The bimodal distributions **do** correspond…” as opposed to “The bimodal distributions correspond…”

These were changed, and we used a grammar checking tool on the entire document.

- There seems to be a mix of active/first person tense (e.g. line 257-265) and passive/third person tense (e.g. line 266-279).

We have removed first person tense from this section.

- Line 71 you say “layout of specific cells”, better to say “spatial organization”

Changed as per reviewer suggestion.

- Line 105 fix ref 13 before period. (in many cases in text reference is after period).

We fixed all references to appear before period.

- Line 162: “Bacteria grow…” is in present tense, but Line 165-166 has “Macrophages will…” which is in future/conditional tense.

We changed these lines to present tense.

- Line:263: data ARE

This was fixed as suggested.

- Line 264 this second part is not clear : “a wide data range to calibrate to at the end time point.” ??

Here we are trying to say the range/spread of the data used in calibration is large. We have adjusted the wording to make this clearer.

- Line 265: “heterogeneous” is redundant.

We removed redundancy as suggested.

- Line 350—could you remove the ability of activated macs to be activated to see if that attenuates?

That is a good suggestion. However, based on the model structure, in our “virtual knock-out” of IFN-γ secretion from CD4+ and CD8+ T cells (section 3.6), we are effectively removing macrophage activation as we have eliminated STAT1 activation. In this case, indeed impaired bacterial control is seen. We made this connection in section 3.6, by mentioning that this double knockout confirms the role of macrophage activation seen in section 3.2.

- The figures don’t gray-scale very well for print - the light-green color for “traditional” are very, very light. Perhaps red or a darker green would be an easy change?

We tried to change the color as suggestion and tested printing it, but this instead made the two groups hard to distinguish. We do agree that the traditional line plots are hard to see in grey-scale, so we instead made the lines thicker to improve visibility.

- The y-axis bars in figure 8 are inconsistently styled between rows 1 and 2.

We understand the concern about the inconsistency. However, Rows 1 and 2 are plotting different outputs and are styled in different ways (log and linear scales, respectively) to best visualize these different outputs.

- Check grammar/revise wording on line 577; it was both an important statement and somewhat difficult to parse.

We revised this statement to make it clearer.

- Line 92 - General modelers may not know what PBMCs are *a priori*.

We defined PBMC at first mention.

- More detail about how the results and predictions of the modeling analysis and how it was performed and the approach(es) used in Figures 5-7 is needed.

We are unsure to which analyses this comment refers. However, assuming that it is referring to the distance metric used in Figures 5 and 7. We have expanded on the approach used in sections 3.3 and 3.5.

- Lines 80-95  can you elaborate what gets put into the “dishes” for the 2-D or 3-D to represent the immune response (cytokines? Cells? Which ones?) A quick list to give an idea would help to distinguish from the biomimetic that follows.

We added a sentence briefly outlining the kinds of cells, bacteria, and extracellular matrix that would be included in these cell culture models.

- Line 101 needs a reference.

We added reference to this line.

- Line 113—this has been observed in PMCID: [7855029](http://www.ncbi.nlm.nih.gov/pmc/articles/PMC7855029), (I see you reference it at line 675)

Thank you for making this connection, we have now made this hypothesis more specific and added a citation to this work earlier in the introduction.

- Lines 80-109 YOU Need to refer to Figure 1! Would have addressed point above

We have added references to Figure 1 into third suggested paragraph.

- Line: 212—‘knockout’ is a colloquial term that doesn't explain what it is ---.suggest that you use the phrase “ virtual gene deletion (i.e. virtual knockout)” to clarify to audience first time – also be sure that it is clear if it is the model in each case that is knocking out or the in vitro system.

We made this change as suggested and clarified in the methods that whenever virtual knockouts are mentioned it is referring to the simulation.

- Line: 223- figure 2 legend should we assume there is no 2-D simulation? Since you only mention 3-D? maybe clarify?

We clarified that what was previously referred to as “2D” is being represented by our traditional model.

- Line: 241 legend 3—I missed where/how the  in vitro dataset shown was  generated/described? Needs methods or clarity. (also line 258 “experimental data at day 6.”

These data are described in section 2.1. We have added a reference to this section to make this connection more clear. We have added a section with a brief description of how calibration was done and what data was used and referenced that as well.

- Line: 514- I was thinking a results table that outlines the different conditions and findings would be cool and helpful.

This is a good suggestion, and we have added Table 2 that summarizes the different conditions and the findings.

- Line 586: the authors acknowledge there is large potential for parameter uncertainty but do not do an uncertainty or sensitivity analysis in this work which is odd..can they clarify? A  sensitivity analysis would certainly formalize many of the results that they predicted and would tandem nicely on the correlations between wet and computer.

We agree that this is a helpful inclusion and have a sensitivity analysis presented in the original model paper. We now mention this uncertainty analysis in the new section on calibration and parameter selection and have added references to these correlations in the results section where appropriate/applicable.

- Also the authors refer to “appendix” throughout. Do they mean “supplement”?

Yes, thank you for this catch. We have updated this throughout the manuscript accordingly.

- Are parameters uniformly sampled? A table of parameters, ranges, distributions, and references for how those distributions were selected would help keep the reader grounded throughout the paper.

We have moved the table of parameters from the supplement into the main text. We have also added a section talking about calibration and parameter selection in more detail.

- It is sometimes difficult to tell whether certain italicized quantities are (input) parameters, intermediate behaviors, or outcomes. For example, I don’t know if *CD8KillProbability* is an independent model input or if this is an emergent quantity internal to the model based on other factors. This is doubly important because for some (*cd4doublingtime*) appears to be the main bifurcation parameter about which the DC-1/DC-2 delineation are based.

We have reviewed the manuscript to confirm that all italicized quantities are parameters. This has been clarified though the addition of Table 1 and a section discussing calibration.

- There was a statement that “Several parameters [show] bimodal distributions” which appears to be a very substantial claim.  This suggests that there’s another mechanism, not represented, that switches between two modes - and whatever those modes are seem to be a major differentiator between DC-1 and DC-2. I find myself wondering “What is the biological interpretation of that bimodal distribution? What is that other mechanism?” Can you comment more about the biological intuition/hypothesis that drives your distinction between DC-1 and DC-2?

This is an excellent point, and something we did not address explicitly before. DC-1 parameter trends include impaired CD8+ T cell behaviors (lower maximum CD8+ T cell generations, lower activated TB-specific CD8+ T cell fraction, lower TB-specific CD8+ T cell fraction, higher CD8+ T cell deactivation probability), improved CD4+ T cell behaviors (higher CD4+ T cell activated lifespan, higher activated TB-specific CD4+ T cell fraction, higher CD4+ T cell IFN and TNF secretion), and some parameters that don’t fit either of these groupings (lower STAT1 span, lower macrophage maximum life span, higher granuloma fraction of diffusion, higher CD8+ kill probability, slightly lower bacterial external doubling time, lower phagocytosis threshold, higher lifespan for activated macrophages). The distributions of the parameters for these two groups can be seen in S1 Fig. As many of these parameters show inverted relationships between CD4+ and CD8+ T cell behaviors, suggesting that the distinction between these groups is which T cell fraction is dominant. These could represent different individuals, similar to results in different mouse strains (62). It could also represent different immune statuses (e.g., people with immunosuppression or cells that are exhausted). We now discuss this in more detail in section 3.1.

- Moreover, it seems like there should be some sort of sensitivity analysis performed to determine the impact of certain parameters (e.g. *cd4doublingtime*) on bacteria count as further quantitative support for the use of that parameter to split the runs into DC1/DC2.  If this is not a reasonable ask, then perhaps you can comment on the computational burden of running these simulations (e.g. if they require a lot of GPU usage, wall time, etc.) Or, perhaps make a clearer statement that a one-at-a-time SA was run - perhaps something that looks like Figure 3, but where you only vary one parameter.

This is a good suggestion to quantify the role of key parameters. However, we do not believe that the impact is solely due to *cd4doublingtime*, rather due to a combination of parameters. We see bimodal distributions in many of the parameters, and a point was arbitrarily chosen between the two peaks of *cd4doublingtime* and used to split the runs. Other parameters could be used to split the runs with equivalent results. These two groups are also found if dividing between the two peaks based on *maximumCD8Generations* at 14.8, *phagocytosisThreshold* at 9.1, *ActivatedCD4IFNSecretion* at 26, or *ActivatedCD4TNFSecretion* at 32. This clarification has now been included in the main text. This also suggests that a one-at-a-time SA using *cd4doublingtime* would not necessarily have a large impact on bacterial count independent of the other varied parameters that appear to compensate for each other.

Furthermore, we have run a global uncertainty analysis that was included in the previous paper, and we now mention this analysis in the text and refer to influence of key parameters where appropriate.

- I am curious to know whether reducing activated macrophage killing in spheroid simulations causes them to return to traditional-like killing rates.

In our “virtual knock-out” of IFN-γ secretion from CD4+ and CD8+ T cells, we are effectively removing macrophage activation as we have eliminated STAT1 activation. In this case, impaired bacterial control is seen. We now emphasize this in section 3.6. These appear to perform more poorly than the traditional control, but this could be due to subsequently impaired CD8+ T cell activation.

- In Figure 8, it seems like there’s about a 100x or more difference between traditional and spheroid CFU counts in the mock/control condition. Does this comment on the quality of traditional *in vitro* culturing? Do you expect the relative fold-changes between spheroid-control and spheroid-knockout to be similar to the fold-changes in the traditional analogue?

We don’t think that this difference comments on the quality of traditional *in vitro* culturing, but it does highlight differences between the traditional and spheroid cultures. These are two different methods to study the same system, one is not necessarily better than the other, but they serve different purposes.

We don’t expect fold changes to be the same for spheroid-control to spheroid-knockout and traditional-control to traditional-knockout as there is an upper limit of bacterial growth both knockouts will approach, which the traditional control is closer to. But we do expect that they will trend in the same direction. We now mention this in section 3.6.

- In paragraph starting line 509, the authors seem to make a substantial claim about the relative roles of CD8+ and CD4+ T cells, specifically that “...a CD4+ T cell dominant response in DC-1 simulations…” However, the DC1/DC2 conditions appear to be defined based on the ability of CD4+ T cells to proliferate. Does this suggest some sort of “compensatory role” that you expect CD8+ T cells to be playing?

This is a good suggestion and we have addressed this in prior comments about differences between DC-1 and DC-2 as well. The model predicts that there is more than one way to match the given experimental data. CD8+ T cells could be compensating for decreased CD4+ T cell proliferation and secretion with increased proliferation and generations. We have added this discussion to this paragraph in section 3.6.

- In section 3.7, the authors claim that “traditional floating” doesn’t fare as well as traditional in some ways. In “traditional floating”, was there a control for any sort of “mean horizontal speed” of cells? Do additional degrees of freedom “slow them down”? I would be interested in the author’s insights as to why traditional-floating had such a different outcome.

This is a good question. There was no control for “mean horizontal speed” of cells, although this is likely slightly different. We hypothesize that the addition of the 3rd dimension as an option for movement effectively reduces the concentration of cells. We see this in Figure 9h and i where the traditional floating cells are slightly farther apart that the equivalent cells in the traditional setup, which is reflected in the macrophage and CD8+ T cell activation. We added this discussion to the paper in section 3.7.

- For the spheroid-gravity simulations that controlled very poorly, were the simulation initial conditions similar to the spheroid in terms of the spatial distribution of T-cells and macrophages? I am left to speculate that the T cells are being “Gravity filtered” out of the simulated *in vitro* granuloma, like in a column filter.

The spheroid gravity simulations start with the cell in similar initial conditions. But, as the movement rules are gravity limited, the cells quickly fall to the bottom of the dish. As the macrophages are added at day 0 and the T cells are added at day 2, we think that the macrophages are forming a compact dome that the T cells then surround. This conserves the layered structure, but it is forced to be denser. Due to the tight packing of the macrophages in this scenario, the T cells infiltrate less and have less opportunity for interaction. We added this discussion to the paper in section 3.7.

**Additional changes to clarify organization:**

Moved references for supplement out of main text into supplement

Formatted SI references

Changed reference to preprint to published paper

**Final additional checks**

PACE for figure (<https://pacev2.apexcovantage.com>)
