# Supplementary material for: Agent-based model predicts that layered structure and 3D movement work synergistically to reduce bacterial load in 3D in vitro models of tuberculosis granuloma
Source: PLoS Comput Biol. 2024 Jul 12;20(7):e1012266. doi: 10.1371/journal.pcbi.1012266 (PMC11288457; doi:10.1371/journal.pcbi.1012266)
Supplement: Attachment — Submitted filename: 2024_06_19_PLOSCB_RtR.docx [file pcbi.1012266.s014.docx]

Reviewer #3: Excellent job addressing concerns. nice job!

Thank you for your comments that helped us improve the manuscript.

Reviewer #4: This revised manuscript by Petrucciani et al. report a series of studies using their recently-described in vitro 3D spheroid granuloma model in order to better understand how specific permutations affect bacterial control. Tuberculous granulomas are complex structures with a network of interactions between multiple immune cell types and they are a key determinant of TB control or progression. Naturally formed granulomas from human or NHP are difficult to isolate and manipulate experimentally. In vitro models offer a tractable platform by which to test specific hypotheses. Here the authors describe a series of elegant simulations using their computational model recently described in PMID 38517920 to test hypotheses about the role of CD4+, CD8+, and macrophages in controlling Mtb within in vitro granulomas. The studies are well conceived and executed and the revised manuscript is very well written. Their in silico approach is hypothesis-generating and will guide the development of more complex in vitro models, inform in vitro and in vivo studies, and advance our understanding of the immune responses needed to protect from Mtb.

Thank you for the positive feedback.

Reviewer #5: I agree with other reviewers in that this manuscript can be a nice contribution to the field. Although I agree with Reviewer 1 in that a very large list of parameters is being `calibrated' based on relatively few data points, the hypotheses and dynamics generated in this study (in silico) have the potential to be tested in the future in vitro/ex vivo/in vivo experiments. In the revised version of the manuscript, the main model hypotheses and used parameter calibration methods have been better explained.

Overall, I believe the authors have successfully addressed all comments from reviewers.

A few relatively minor points:

- I would recommend the authors to refer to Figure 2 in (15) where a nice overall description of the model is given.

We have added this reference as suggested.

- I believe that Table 1 is currently very much a reproduction of Table 1 in (15). I imagine there is no issue since the copyright of (15) is with the authors, and I can see the benefit of including this table in this manuscript. However, I would recommend making this clear and citing such table.

We have made it clear that this table is adapted from (15) and added a citation.

Moreover, a list of parameters with values without a clear description of what each parameter represents is not necessarily useful, and makes the interpretation of results (corresponding to specific parameter values) difficult for the reader. Can the authors refer to a clear comprehensive glossary of parameter definitions from somewhere? Either from the Supplementary, the Github repository or Ref (15).

We have added a table with elongated descriptions of each parameter to the supplement.

- Please make sure that resolution of all images is good enough.

We have checked our images and updated them accordingly.

- I would strongly recommend that key data is provided numerically, rather than just as figures (e.g. boxplots), for reproducibility purposes. Similarly, key summary statistics of calibrated parameters would facilitate reproducibility.

Numerical versions of key data, raw data, and parameter values can be found in the .mat files and the .m processing scripts included in the Zenodo repository (DOI: 10.5281/zenodo.10035530).
